# BioCaster in 2021: automatic disease outbreaks detection from global news media

**DOI:** 10.1093/bioinformatics/btac497

**Published:** 2022-07-28

**Authors:** Zaiqiao Meng, Anya Okhmatovskaia, Maxime Polleri, Yannan Shen, Guido Powell, Zihao Fu, Iris Ganser, Meiru Zhang, Nicholas B King, David Buckeridge, Nigel Collier

**Affiliations:** Modern and Medieval Languages and Linguistics, University of Cambridge, Cambridge CB3 9DA, UK; School of Computing Science, University of Glasgow, Glasgow G12 8QQ, UK; School of Population and Global Health, McGill University, Montreal H3A 1G1, Canada; Département d’anthropologie, Université Laval, Québec G1V 0A6, Canada; School of Population and Global Health, McGill University, Montreal H3A 1G1, Canada; School of Population and Global Health, McGill University, Montreal H3A 1G1, Canada; Modern and Medieval Languages and Linguistics, University of Cambridge, Cambridge CB3 9DA, UK; School of Population and Global Health, McGill University, Montreal H3A 1G1, Canada; Modern and Medieval Languages and Linguistics, University of Cambridge, Cambridge CB3 9DA, UK; School of Population and Global Health, McGill University, Montreal H3A 1G1, Canada; School of Population and Global Health, McGill University, Montreal H3A 1G1, Canada; Modern and Medieval Languages and Linguistics, University of Cambridge, Cambridge CB3 9DA, UK

## Abstract

**Summary:**

BioCaster was launched in 2008 to provide an ontology-based text mining system for early disease detection from open news sources. Following a 6-year break, we have re-launched the system in 2021. Our goal is to systematically upgrade the methodology using state-of-the-art neural network language models, whilst retaining the original benefits that the system provided in terms of logical reasoning and automated early detection of infectious disease outbreaks. Here, we present recent extensions such as neural machine translation in 10 languages, neural classification of disease outbreak reports and a new cloud-based visualization dashboard. Furthermore, we discuss our vision for further improvements, including combining risk assessment with event semantics and assessing the risk of outbreaks with multi-granularity. We hope that these efforts will benefit the global public health community.

**Availability and implementation:**

BioCaster web-portal is freely accessible at http://biocaster.org.

## 1 Introduction

Disease outbreaks cause widespread suffering and have contributed to major health inequalities across the world (Chowkwanyun and Reed, 2020). Traditional disease surveillance systems rely on data from networks of laboratories and human experts, which might be unavailable in real time, uneven in geographic coverage and tuned to specific diseases. Digital Disease Surveillance (DDS) complements traditional surveillance systems and is used worldwide to detect public health threats resulting from the rapid spread of infections, as illustrated by the ongoing COVID-19 pandemic. Existing DDS systems, such as BioCaster (Collier *et al.*, 2008), Medisys (https://medisys.newsbrief.eu) (Steinberger *et al.*, 2008), EpiCore (https://epicore.org) (Olsen, 2017) and HealthMap (https://www.healthmap.org) (Freifeld *et al.*, 2008) use streaming news data to overcome some of the limitations of traditional systems, providing a critical supplement to public health surveillance. BioCaster was launched in 2008 and used ontology-based text mining techniques to detect early disease outbreaks from open news sources. Meanwhile, some nonprofit and commercial DDSs, such as International SOS (https://www.internationalsos.com) and Bluedot (https://bluedot.global/), spend massive efforts in quickly responding to these outbreaks and reducing health or security incidents. However, these DDS works paid little attention on equality considerations that arise from biases in the data. Moreover, given that streaming news data are high-volume, multilingual, high-velocity and potentially biased, DDS must incorporate the latest Natural Language Processing (NLP) techniques to refine predictive models for future outbreak scenarios, so that more accurate response can be made.

Recent Artificial Intelligence (AI) technologies, such as deep learning and NLP, have proven to be effective methodologies to enhance various healthcare applications by using the vast amount of data available from bio-medical studies (Gu *et al.*, 2021). Following a 6-year break, we have re-launched our BioCaster system in 2021 by integrating the latest AI techniques to achieve a step-change in real-time disease outbreak understanding and detection. In particular, our new BioCaster adopts an interdisciplinary approach, combining expertise from three disciplines—computer science, epidemiology and bioethics—to develop novel deep learning and NLP models adapted to the structurally complex data and objectives of global epidemic surveillance. Whilst retaining the original benefits that the system provided of logical reasoning and early detection for infectious disease outbreaks, these neural NLP models equip our new system with the ability to incorporate domain knowledge. Moreover, a more interactive user interface allows users to customize the view according to the geographic location, disease and language from a temporal perspective. Our system currently processes thousand news reports per day, automatically detects outbreak reports, extracts key data of epidemiological relevance and then allows users to interactively visualize news statistics and outbreaks through a web-based user interface according to their area of interest in real time.

## 2 Biocaster

### 2.1 Architecture

**Fig. 1.**
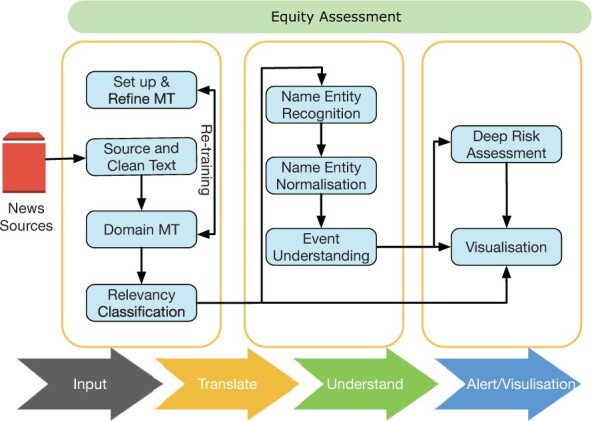
The information flow through our BioCaster system

The goal of our BioCaster is to achieve fully automated real-time media monitoring based on streaming news data. Our system achieves this goal by processing news data in four steps (Fig. 1): (i) the first step (**Input**) integrates news data from a variety of news sources, such as Google news and RSS news feeds; (ii) the second step (**Translate**) is a multi-functional module responsible for translating the various news documents from 10 languages into English and identifying the biomedicine-related news documents through neural models; (iii) during the third step (**Understand**), the system then tries to convert these documents into structured event semantics by using a mixture of rule-based methods, named entity recognition, and entity normalization techniques; and (iv) in the final step (**Alert**), the alerts and their related documents, counts and alert scores are plotted onto a public visualization portal implemented by Kibana (https://www.elastic.co/kibana/).

### 2.2 Key features

To systematically upgrade the system with the rapidly evolving technologies, we developed novel deep neural models, adopted new assessment methodologies and deployed a new user interface to upgrade our system, where these new upgrades are able to improve the system in terms of accuracy, reliability and interactivity. In particular, the new system differs from the old ones in four key features: multilingual translation, deep neural techniques, time series user interface and equity assessment.

**Multilingual translation.** Our BioCaster acquires news from various global news media over 10 languages, including two low resource languages (i.e. Swahili and Farsi). Language Weaver Edge, a translation portal provided by our partner RWS Language Weaver (https://www.rws.com/language-weaver/edge/), has been deployed to translate news from world languages into English for further processing.

**Deep neural techniques.** Deep neural NLP models have been developed to fuse text and knowledge graphs. In particular, we adopt the pre-trained PubMedBERT and SapBERT (https://github.com/cambridgeltl/sapbert) models (Liu *et al.*, 2021) for relevance classification and entity linking, respectively. To support learning from small labeled data, an extensive analysis of contextualized representation learning methods has been performed to fuse together text and expert knowledge from UMLS (https://www.nlm.nih.gov/research/umls/) ontologies (Meng *et al.*, 2021).

**Time series user interface.** A web-based user interface has been deployed by constructing a cloud-based Kibana server to visualize the news statistics and outbreak alerts from our models. This new user interface combines a dashboard with a geographic map, tendency charts and tables, where the filtering and visualization features bring the users an interactive interface for accessing news, alerting and statistics from a temporal perspective (see http://biocaster.org/ for the user interface).

**Equity assessment.** Our team undertook a comprehensive equity assessment to identify potential problems that might arise during the design and implementation of this DDS. Three clusters of equity concerns were identified: (i) the use of systematically biased data arising from media sources with uneven coverage of different diseases, geographic areas, and social groups; (ii) the potential for exacerbating extant health inequalities through uneven coverage and/or access to information; and (iii) identifying and mitigating potential unintended consequences, such as misuse of DDS information. To address concerns (i) and (iii), we are designing and will implement a qualitative flagging system to alert users to potential biases arising from uneven media coverage—for example, lower coverage during weekends and holidays, and lower coverage in countries with less press freedom. To address concern (ii), we are conducting a qualitative needs assessment as part of an effort to address the needs of users from underserved communities.

## 3 Conclusion

The promise of BioCaster lies in its ability to understand the mass of unstructured data about disease outbreak events from open news sources. While the new deployed deep neural techniques have improved its usability and capability to process large amount of news data and detect disease outbreaks in real time, there remains room for further improvements.

**Limitations:** (i) Our current system deploys count-based alert metrics for risk assessment, which limits its ability to cope with negations, such as those reports showing a sharp decrease of cases. This could be improved by combining risk assessment with event semantics to more fully integrate representation learning with event trend understanding. (ii) Our current system applies a rule-based event extraction approach with a fixed granularity of event definition, which lacks in capturing the evaluating aspects of events. Modeling outbreak events with multi-granularity could better understand the spatial and temporal evolution of disease outbreaks, which is our future work. (iii) Our current system focuses on only the traditional media, evaluating the potential of using non-traditional media sources such as internet memes is another area of future work.

## Supplementary Material

btac497_Supplementary_Data
