# Supplementary material for: BioCaster in 2021: automatic disease outbreaks detection from global news media
Source: Bioinformatics. 2022 Jul 28;38(18):4446–8. doi: 10.1093/bioinformatics/btac497 (PMC9477518; doi:10.1093/bioinformatics/btac497)
Supplement: btac497_Supplementary_Data [file btac497_supplementary_data.pdf]

BioCaster in 2021: Automatic Disease Outbreaks Detection from Global News Media

Supplementary Information

Zaiqiao Meng, Anya Okhmatovskaia, Maxime Polleri
Yannan Shen, Guido Powell, Zihao Fu, Iris Ganser, Meiru Zhang
Nicholas B. King, David Buckeridge, Nigel Collier

1 Relevance Classification

Relevance classification is one of tasks at the first stage of our BioCaster system. The goal of this task is to identify disease outbreak related news reports from all the collected news documents, then these positive disease outbreak related news will be passed into the next stage for event understanding. We evaluate a number of document classification models on two outbreak related datasets and find that fine-tuning pre-trained language models (particularly the biomedical specific ones) yields higher performance than traditional machine learning models such as SVM and decision tree.

1.1 Training Datasets

Two datasets, namely the LitCovid and the ProMed-26k datasets are used to evaluate and compare various document classification models. Table 1 shows the statistics of the two used datasets.

- **LitCovid**. This is a multi-class document classification dataset about 2019 novel Coronavirus, which is collected from the PubMed articles [6]. The task of this dataset is to classify these articles into 8 topic labels: Prevention, Treatment, Diagnosis, Mechanism, Case Report, Transmission, Forecasting and General. We use accuracy and the micro F1 scores as the evaluation metric for this dataset.

- **ProMed-26k**. This is a binary document classification dataset collected from various source, including ProMed-mail [1], Reuters news[2] and BBC news[3], where ProMed-mail news are regarded as positive documents and the other two are regarded as the negative ones. The goal of this dataset is to identify which news reports are disease outbreak related ones, and

[1] https://promedmail.org/
[2] http://disi.unitn.eu/moschitti/corpora.htm
[3] https://www.kaggle.com/c/learn-ai-bbc

the trained model is also used as our document classifier in the BioCaster system. We use accuracy and the F1 scores as the evaluation metric for this dataset.

	**LitCovid**	**ProMed-26k**
# of Classes	8	2
# of Articles	23,038	25956
Avg. sentences	74	12
Avg. tokens	1,399	384
Total # of tokens	32,239,601	9,975,402

Table 1: Dataset statistics for the *LitCovid* and *ProMed-26k* Datasets.

1.2 Document Classification Models

Two types of document classification models, namely the traditional machine learning models and the pre-trained language models, are used to evaluate the relevance classification task under our two datasets. In particular, for traditional machine learning models we use the C4.5 decision tree algorithm, the SVM algorithm and their variants with chi-squared test feature selection (i.e. C4.5+Chi2 and SVM+Chi2) [14]; for pre-trained language models we use BERT [8], BioBERT [9], SciBERT [2], SapBERT [12] and PubMedBERT [5] models. These traditional machine learning models are trained based on the TFIDF feature of the document text.

1.3 Results

Table 2 shows the comparison result of document classification models. In general, we see that pre-trained language models perform better than the traditional machine learning models (e.g. SVM and C4.5) even they have equipped with the chi-squared test feature selection approaches. We also see that in both the datasets, the performance values of these pre-trained models are quite close on all the evaluate metrics, and all of them achieve a ∼99% accuracy and F1 scores, which means they are all the suitable models for our BioCaster system. In particular, the PubMedBERT [5] model performs the best among all these models on both of the datasets, which is the main reason that our BioCaster applies the PubMedBERT model for the relevance classification task.

2 Entity Linking

In the second stage of our system, we extract the event semantics of each documents, which requires solving some additional NLP tasks, such as named entity recognition and entity linking. The entity linking task is to link the different surface forms of same entity to a unified entity ID and name in the knowledge

Model	LitCovid		ProMed-26k	
	Acc.	Micro F1	Acc.	F1
**SVM**	-	-	98.96	96.49
**C4.5**	-	-	96.53	88.10
**SVM+Chi2**	-	-	98.42	94.65
**C4.5+Chi2**	-	-	96.92	89.36
**BERT-base**	74.18±0.66	85.61±0.47	99.13±0.08	99.49±0.05
**SciBERT**	75.30±1.53	86.47±0.57	99.02±0.08	99.43±0.09
**BioBERT**	75.58±0.52	85.87±0.02	99.04±0.15	99.43±0.09
**SapBERT**	75.75±0.44	86.62±0.36	98.95±0.08	99.38±0.05
**PubMedBERT**	**76.04±0.33**	**86.63±0.19**	**99.19±0.10**	**99.53±0.06**

Table 2: Comparison of performance (*mean* ± *standard deviation*) over 3 runs. **Bold** denotes the best performing result comparing with others.

graph (e.g. SNOMED CT [4], that is used in our system). In particular, we propose a novel entity representation model based on the pre-trained language model, called SapBERT [12], which is able to achieve the state-of-the-art performance in the biomedical entity linking task. Our SapBERT model is build based on the self-alignment pre-training over the UMLS knowledge graph, which is the largest biomedical ontologies, containing a comprehensive collection of biomedical synonyms in various forms. The UMLS collection contains **4M+ concepts** and **10M+ synonyms**, stemming from **over 150 controlled vocabularies**. Our SapBERT applies a metric learning framework that self-aligns synonym representations belonging to the same UMLS concept. More details about the SapBERT model can be found in our published article [12].

We use 6 different biomedical entity linking datasets, including 4 in the scientific domain (NCBI [4] (D1); BC5CDR-d (D2) and BC5CDR-c (D3), [10]; MedMentions (D4), [13]) and 2 in the social media domain (COMETA (D5), [1] and AskAPatient (D6), [11]) to evaluate the performance of our SapBERT model. We test our SapBERT under 3 purely pre-trained models, i.e. BERT [8], BioBERT [9] and PubMedBERT [5], with (*BERT+SapBERT) denoting these model that are self-supervisedly pre-trained under the UMLS concepts. Note that our model is a purely unsupervised approach, and can also be applied in any supervised approaches that use pre-trained language models. Therefore, we also evaluate our approach under a state-of-the-art supervised approach BioSyn [15].

Table 3 shows the evaluation result based on the accuracy metric. In general, we find that our SapBERT improves all the three pre-trained models with large gains. Comparing with the state-of-the-art supervised approach, our SapBERT further can improve the BioSyn model, achieving a new state-of-the-art performance.

[4] https://www.snomed.org/

domain→	Scientific				Social Media	
model↓, data set→	D1	D2	D3	D4	D5	D6
BERT	67.6	81.4	79.8	39.6	38.2	40.4
+ SapBERT	91.6	92.7	96.1	52.5	68.4	59.5
BioBERT	71.3	79.8	74.0	24.2	41.4	35.9
+ SapBERT	91.0	93.3	95.5	97.6	72.4	63.3
PubMedBERT	77.8	89.0	93.0	43.9	42.5	46.8
+ SapBERT	92.0	93.5	96.5	50.8	70.5	65.9
BioSyn	91.1	93.2	96.6	OOM	82.6	71.3
+ (init. w/) SapBERT	**92.5**	**93.6**	**96.8**	OOM	**87.6**	**77.0**

Table 3: Accuracy performance comparison of different models on the entity linking task. OOM denotes that the model encounters the out-of-memory issue. **Bold** denotes the best performing result comparing with others.

3 Risk Assessment Evaluation

We integrate various Early Aberration and Reporting System (EARS) algorithms, including C2, C3 and W2, which have been widely used in many EARS of the public health community [7]. In particular, our current BioCaster visualises the alerts using the C2 metric as our risk assessment metric, according to our prior evaluation [3]. C2 is calculated based on the news report counts of the same event on the day basis and over a baseline period:

$$S_t = \max\left(0, \left(C_t - (\mu_t + k\sigma_t)\right) / \sigma_t\right), \tag{1}$$

where C_t is number of news reports on the target day t, μ_t and σ_t are the mean and standard deviation of the counts during the baseline period. In our system, we set the baseline period to 14 days and $k = 1$. Our BioCaster triggers an alert of outbreak when the $S_t >= 1$.

References

[1] M. Basaldella, F. Liu, E. Shareghi, and N. Collier. COMETA: A corpus for medical entity linking in the social media. In *Proceedings of the 2020 Conference on Empirical Methods in Natural Language Processing (EMNLP)*, pages 3122–3137, Online, Nov. 2020. Association for Computational Linguistics.

[2] I. Beltagy, K. Lo, and A. Cohan. SciBERT: A pretrained language model for scientific text. In *EMNLP-IJCNLP*, pages 3606–3611, 2019.

[3] N. Collier. Towards cross-lingual alerting for bursty epidemic events. *Journal of Biomedical Semantics*, 2(5):1–11, 2011.

[4] R. I. Doğan, R. Leaman, and Z. Lu. NCBI disease corpus: a resource for disease name recognition and concept normalization. *Journal of Biomedical Informatics*, 47:1–10, 2014.

[5] Y. Gu, R. Tinn, H. Cheng, M. Lucas, N. Usuyama, X. Liu, T. Naumann, J. Gao, and H. Poon. Domain-specific language model pretraining for biomedical natural language processing. *arXiv preprint arXiv:2007.15779*, 2020.

[6] B. J. Gutiérrez, J. Zeng, D. Zhang, P. Zhang, and Y. Su. Document classification for covid-19 literature. In *Findings of the Association for Computational Linguistics: EMNLP 2020*, pages 3715–3722, 2020.

[7] L. Hutwagner, W. Thompson, G. M. Seeman, and T. Treadwell. The bioterrorism preparedness and response early aberration reporting system (ears). *Journal of Urban Health*, 80(1):i89–i96, 2003.

[8] J. D. M.-W. C. Kenton and L. K. Toutanova. Bert: Pre-training of deep bidirectional transformers for language understanding. In *Proceedings of NAACL-HLT*, pages 4171–4186, 2019.

[9] J. Lee, W. Yoon, S. Kim, D. Kim, S. Kim, C. H. So, and J. Kang. BioBERT: a pre-trained biomedical language representation model for biomedical text mining. *Bioinformatics*, 36(4):1234–1240, 2020.

[10] J. Li, Y. Sun, R. J. Johnson, D. Sciaky, C.-H. Wei, R. Leaman, A. P. Davis, C. J. Mattingly, T. C. Wiegers, and Z. Lu. BioCreative V CDR task corpus: a resource for chemical disease relation extraction. *Database*, 2016, 2016.

[11] N. Limsopatham and N. Collier. Normalising medical concepts in social media texts by learning semantic representation. In *Proceedings of the 54th Annual Meeting of the Association for Computational Linguistics*, pages 1014–1023, 2016.

[12] F. Liu, E. Shareghi, Z. Meng, M. Basaldella, and N. Collier. Self-alignment pretraining for biomedical entity representations. In *Proceedings of the 2021 Conference of the North American Chapter of the Association for Computational Linguistics: Human Language Technologies*, pages 4228–4238, 2021.

[13] S. Mohan and D. Li. MedMentions: A large biomedical corpus annotated with UMLS concepts. In *Automated Knowledge Base Construction*, 2018.

[14] A. Moh'd A Mesleh. Chi square feature extraction based svms arabic language text categorization system. *Journal of Computer Science*, 3(6):430–435, 2007.

[15] M. Sung, H. Jeon, J. Lee, and J. Kang. Biomedical entity representations with synonym marginalization. In *Proceedings of the 58th Annual Meeting of the Association for Computational Linguistics (ACL)*, pages 3641–3650, Online, July 2020. Association for Computational Linguistics.
